# Classification of clinical skin lesions with double-branch networks

**DOI:** 10.3389/fmed.2023.1114362

**Published:** 2023-06-09

**Authors:** Hui Wang, Qianqian Qi, Weijia Sun, Xue Li, Chunli Yao

**Affiliations:** ^1^College of Computer Science and Engineering, Changchun University of Technology, Changchun, China; ^2^Department of Dermatology, The Second Hospital of Jilin University, Changchun, China

**Keywords:** clinical images, skin classification, medical image classification, deep learning, dermatology

## Abstract

**Introduction:**

Malignant skin lesions pose a great threat to the health of patients. Due to the limitations of existing diagnostic techniques, such as poor accuracy and invasive operations, malignant skin lesions are highly similar to other skin lesions, with low diagnostic efficiency and high misdiagnosis rates. Automatic medical image classification using computer algorithms can effectively improve clinical diagnostic efficiency. However, existing clinical datasets are sparse and clinical images have complex backgrounds, problems with noise interference such as light changes and shadows, hair occlusions, etc. In addition, existing classification models lack the ability to focus on lesion regions in complex backgrounds.

**Methods:**

In this paper, we propose a DBN (double branch network) based on a two-branch network model that uses a backbone with the same structure as the original network branches and the fused network branches. The feature maps of each layer of the original network branch are extracted by our proposed CFEBlock (Common Feature Extraction Block), the common features of the feature maps between adjacent layers are extracted, and then these features are combined with the feature maps of the corresponding layers of the fusion network branch by FusionBlock, and finally the total prediction results are obtained by weighting the prediction results of both branches. In addition, we constructed a new dataset CSLI (Clinical Skin Lesion Images) by combining the publicly available dataset PAD-UFES-20 with our collected dataset, the CSLI dataset contains 3361 clinical dermatology images for six disease categories: actinic keratosis (730), cutaneous basal cell carcinoma (1136), malignant melanoma (170) cutaneous melanocytic nevus (391), squamous cell carcinoma (298) and seborrheic keratosis (636).

**Results:**

We divided the CSLI dataset into a training set, a validation set and a test set, and performed accuracy, precision, sensitivity, specificity, f1score, balanced accuracy, AUC summary, visualisation of different model training, ROC curves and confusion matrix for various diseases, ultimately showing that the network performed well overall on the test data.

**Discussion:**

The DBN contains two identical feature extraction network branches, a structure that allows shallow feature maps for image classification to be used with deeper feature maps for information transfer between them in both directions, providing greater flexibility and accuracy and enhancing the network's ability to focus on lesion regions. In addition, the dual branch structure of DBN provides more possibilities for model structure modification and feature transfer, and has great potential for development.

## 1. Introduction

Skin lesions are a common proliferative disease of the skin with many types, clinically classified as benign and malignant lesions (1). Benign skin lesions grow more slowly and do not metastasize or spread to local structures or distant parts of the body and usually do not require treatment (2). Malignant skin lesions have the risk of invading surrounding tissues and organs and metastasis, and early diagnosis and timely treatment can improve the cure rate and survival rate. Therefore, it is important to find a efficient and accurate diagnostic method for early skin malignancies. With the rise of artificial intelligence (AI), computer diagnosis of skin diseases has become possible (3, 4). AI has great potential to improve the diagnosis of dermatologists and to promote the construction and development of the discipline. Artificial intelligence systems can support dermatologists in their daily clinical practice, physicians can also provide clinical information to system to improve the correct diagnosis rate of the AI.

So far, there are still many scholars studying about skin classification to obtain higher accuracy, while Khouloud et al. (5) proposed a deep learning model for detecting melanoma, which consists of two parts, a segmentation network called W-Net, and a classification network Inception ResNet, and experiments showed that the W-Net has excellent performance in segmentation and classification with higher accuracy. Benyahia et al. (6) investigated the efficiency of using 17 common pre-trained convolutional neural network architectures as feature extractors and 24 machine learning classifiers and found that DenseNet201 (7) combined with Fine KNN or Cubic SVM achieved the best accuracy on the ISIC2019 dataset and PH2 dataset. Popescu et al. (8) proposed a skin lesion classification system based on deep learning techniques and collective intelligence, analyzing the performance of nine classification networks to obtain a weight matrix to make final and more accurate decisions related to prediction based on the associated weights of each network output. Hasan et al. (9) proposed an optimized color feature (OCF) for skin lesion segmentation and a deep convolutional neural network (DCNN) based classification of skin lesions. A hybrid technique was also proposed to eliminate artifacts and improve lesion contrast. Features are extracted using the DCNN-9 model and fused with OCFs by a parallel fusion method. Finally, a high-ranking feature selection technique based on normal distribution is used to select the most robust features for classification.

Dermatoscopy, also known as skin surface transillumination microscopy, is gradually being promoted and applied by dermatologists everywhere as a new adjunct to clinical examination and diagnosis in dermatology, due to its ease of use, non-invasiveness, and improved diagnostic accuracy. Similarly, most current classification models use dermatoscopic images as the main training set, as dermatoscopic images contain less noise and have better training results. Less research has been done on clinical images taken by digital cameras in the traditional way. So we construct a clinical dataset and designs a double-branch network structure for clinical images, which contains the original network branch and the fusion network branch, and it supports the fusion of feature maps between different levels for passing, thus improve the feature extraction capability of the network. The main contributions are summarized as follows.

1. We constructed a new dataset, CSLI (Clinical Skin Lesion Images), which contains more than 3,000 clear clinical skin medical images of six common skin disease categories.
2. We proposed a double-branch network structure DBN, which can be used on different Backbone, and its main purpose is to achieve the transfer and fusion between shallow and deep feature maps.
3. We proposed CFEBlock and FusionBlock, where CFEBlock achieves the extraction of common features from the feature maps of adjacent layers by the operation of convolutional dot product addition, and FusionBlock is responsible for efficiently combining the common feature maps obtained from the common feature extraction part with the feature maps of the fusion network branches.
4. We tested the proposed DBN on the CSLI and HAM10000 dataset and compared it with other networks, and finally concluded that the DBN performed better overall.

The remainder of the paper is structured as follows: in Section 2 we summarize related work, and in Section 3 we describe the preparation of the dataset in question and the setup of the DBN. In Section 4, we conduct experiments on the CSLI dataset and compare the effectiveness of the DBN with baseline. In Section 5, we discuss the implications of the results of this work, summarize and indicate directions for future work.

## 2. Related work

In recent years, convolutional neural networks (CNN) based approaches have made impressive advances and developments in computer vision. At the core of artificial intelligence-aided diagnosis of skin lesions is the automatic computer classification of skin lesions images, and the task of image classification is a very general problem: any problem that requires distinguishing between different associated images can fall into this category. Today, an increasing number of scholars are using deep learning-based methods for skin lesions classification.

Han et al. (10) used CNN to classify up to 134 diseases and showed that CNN was more effective than dermatologists in analyzing blurred, indistinguishable images. Hasan et al. (9) proposed an automated dermoscopic SLC framework called Dermoscopic Expert (DermoExpert) and performed experiments on ISIC datasets. The method combines preprocessing and hybrid Convolutional Neural Network (hybrid-CNN). In the proposed preprocessing, lesion segmentation, enhancement (based on geometry and intensity) and category rebalancing (penalizing the loss of most categories and merging additional images into few categories) are applied. While ResNet (11), one of the dominant classification models in convolutional neural networks, remains the preferred backbone network for a large number of researchers, Han et al. (12) used the ResNet-152 model to classify clinical images of 12 dermatological diseases. Toğaçar et al. (13) proposed an attention mechanism based on ResNet50 adding to the network structure SE-ResNet50 and SE-ResNeXt50, the algorithm of this study showed better results in f1score metrics, surpassing non-dermatologists, with accuracy comparable to that of dermatologists.

Lightweight network models for skin disease classification is also one of the important research directions, which can be embedded in mobile devices, allowing patients to get more accurate test results before the doctor diagnoses them and the doctor can refer to the results afterwards. Toğaçar et al. (13) proposed a model based on autoencoder, impulse neural network and convolutional neural network, which uses the classification network of MobilenetV2 (14), which can greatly save the number of parameters and make it applicable to smartphones, while the autoencoder and impulse neural network compensate for the low accuracy of MobilenetV2. Srinivasu et al. (15) added an LSTM (16) mechanism to MobileNetV2, which on the HAM10000 dataset to achieve 85% accuracy and applied the model to mobile phones. Iqbal et al. (17) designed a lightweight skin classification network, CSLNet, which has high efficiency and performance, the algorithm proposed in this study outperformed the state-of-the-art algorithm on the ISIC dataset.

In addition to using methods, many researchers have combined current hot technologies and others to classify images of skin diseases. Reinforcement learning is a class of frameworks for learning, prediction, and decision making that interacts with the environment and is an algorithm for sequential problems. Compared to supervised learning, reinforcement learning considers long-term payoffs, and this long-term perspective is crucial to find the optimal solution. For example, Akrout et al. (18) introduced reinforcement learning techniques and combined them with CNN to propose a Question Answering (QA) model, which improved the classification confidence and accuracy of a visual symptom checker, while reducing the average number of questions asked. Federated learning can carry out efficient machine learning frameworks among multiple participants or computational nodes while meeting the requirements of user privacy protection, data security, and government regulations. Bdair et al. (19) proposed a semi-supervised federation learning approach and proposed Peer Anonymization (PA) technique to improve privacy and reduce communication cost while improving performance. Knowledge distillation is a method of compressed models, consisting of a teacher network and a student network, which enables the student network to effectively learn the generalization ability of the teacher network by learning the output of the complex and highly generalized teacher network through the streamlined and small parametric student network. Van Molle et al. (20) used knowledge distillation techniques to train deep learning networks and applied them to skin disease classification with good result. Transformer is a model that uses attention mechanism to improve the training speed, which is widely used in natural language processing, and a recent study confirmed its great potential in computer vision field as well, Shamshad et al. (21) provided a detailed summary of the application of Transformer in medical field. Wu et al. (22) cited natural language Transformer technique in analysis combined with classification networks on full-field digital slices, and experimental results indicated that the method outperformed other full-field digital slicing methods. In addition to model and technical innovations, combining medical knowledge to analyze disease categories is also a new direction, for example, Kinyanjui et al. (23) proposed a method to estimate skin color in a benchmark dataset of dermatological diseases and investigated whether model performance depends on this metric. Pacheco and Krohling (24) used metadata (e.g., patient's age, gender, etc.) to aid in classification. They used five different networks and added their proposed metadata processing block, which led to improved classification results of the network.

Clinical images are more challenging to classify than dermoscopic images due to their off-center location of lesions, high noise, and susceptibility to light, which leads to a more challenging classification of clinical images. In clinical skin classification, good classification results have been achieved using a combination of segmentation and classification networks, but the difficulty in labeling the lesion regions have become major problems for the two-stage model. Whereas, the approach using only classification networks does not require additional training of segmentation models, most of the existing skin classification networks are one-way down the process, which leads to the following problems. (1) Feature maps can only be transferred from shallow to deep layers, while deep features cannot be transferred upward. Usually deep learning networks contain richer contour and texture information in the shallow feature maps due to the perceptual field limitation, while the deeper features contain more semantic and location information. The inability of the deep layer to propagate upward leads to possible bias in the location of the image of interest at the shallow layer thus leading to a decrease in accuracy. (2) The one-way network structure is less flexible and cannot be easily modified or modified to add the complete ImageNet pre-training weights to the backbone, which affects the training effect. In summary, this paper proposes a double-branch network model for automatic classification of skin diseases.

## 3. Methods

### 3.1. Dataset construction

The CSLI dataset contains two parts, the data collected from January 2018 to June 2022 from dermatology outpatients at Department of Dermatology of the Second Hospital of Jilin University and the open clinical dataset PAD-UFES-20 from the Federal University of Espirito Santo (25).

The collection was mainly done by a number of experienced dermatologists at the Second Hospital of Jilin University, and the dataset used the pathological diagnosis of the lesion as the real label for the collected images. The dataset contained 1,063 patients and 1,063 images of five diseases: basal cell carcinoma of skin (BCC), squamous cell carcinoma (SCC), seborrheic keratosis (SEK), malignant melanoma (MEL), and melanocytic nevus of skin (NEV). The equipment used for the capture is a professional HD digital camera. We focus on the key areas of the lesion as the center of the shot, adjusting the shooting distance to the exact size of the lesion and taking multiple images from different angles against a solid color background.

Data cleaning and labeling was done by several dermatologists. The labeling of each image we acquired was confirmed by biopsy to ensure the accuracy of the labels. In terms of validity, we performed rigorous data cleaning of the acquired image data, eliminating blurred, duplicate, low-quality images. For multiple images of lesions taken from each patient, we kept only one image of the best quality as a sample of the dataset, after which we further cropped these images to crop out the lesion areas to obtain the precise skin lesion areas that would constitute the dataset used for training. We perform center cropping on the collected images mainly for the following two reasons: (1) The lesions in the PAD-UFES-20 images are basically located in the positive center, and using center cropping can make the sample distribution more uniform. (2) Since there are images with small lesions and large sizes in the clinically taken images, scaling such images before performing network training will cause the lesions with a small percentage of the original area to almost disappear, which is a bad learning sample for the model is a bad learning sample, thus affecting the model's ability to discriminate the disease.

The PAD-UFES-20 dataset has 1,373 patients, 1,641 skin lesions, and 2,298 images. The dataset contains a total of six categories of images, adding one more category of actinic keratosis (ACK) to the five diseases mentioned above. Since the images in this dataset are collected using different smartphone devices they present different resolutions, sizes, and lighting conditions, and all images were stored in PNG file format.

**Figure 1:**
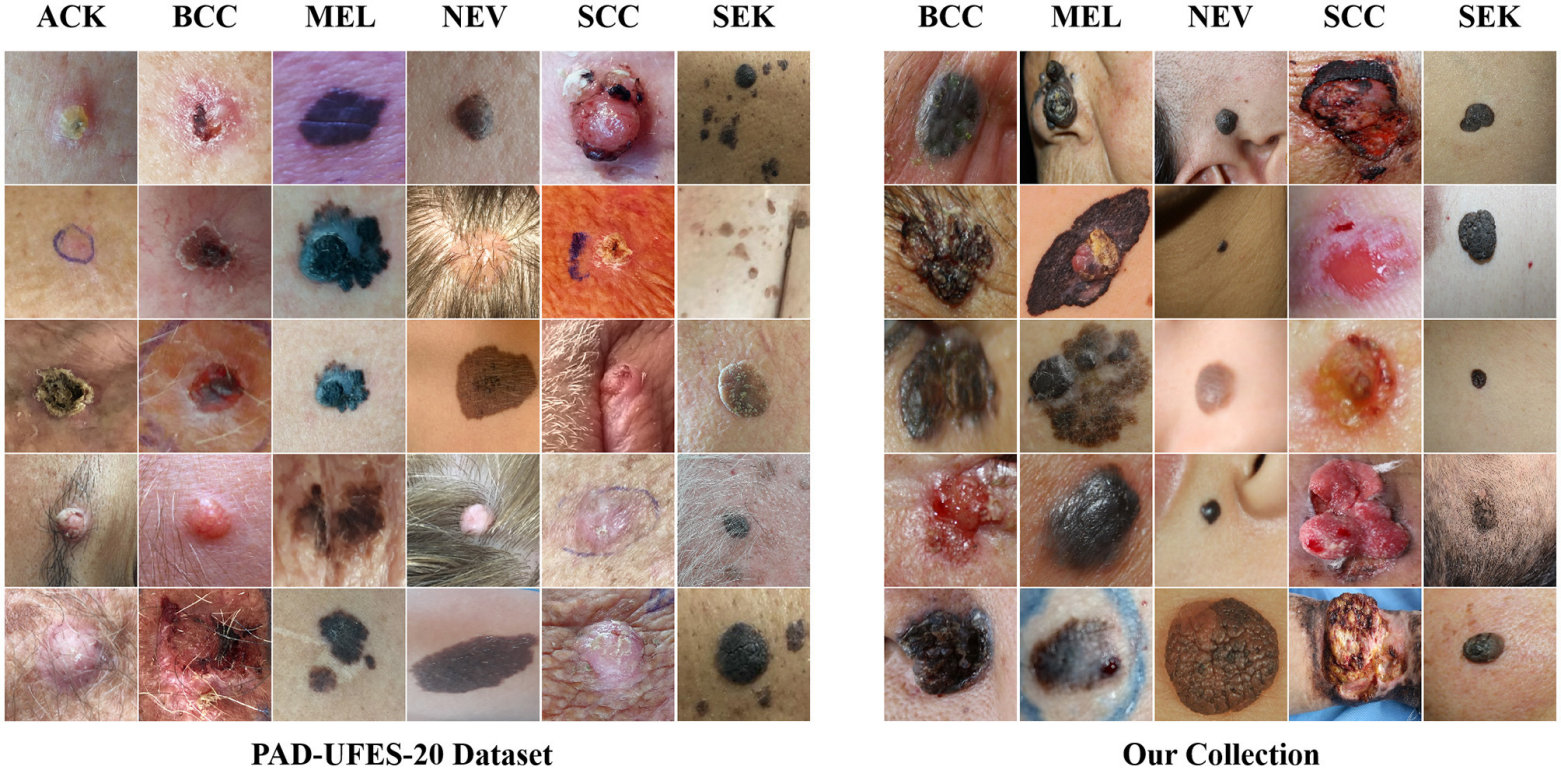
The CSLI dataset.

The CSLI dataset is available from the corresponding author upon reasonable request. Table 1 shows the detailed number of disease samples for each category in the two-part dataset. Figure 1 shows a partial sample of the CSLI dataset.

**Table 1:** Number of disease samples in each category of the data set.

**Disease category**	**PAD-UFES-20**	**Our collection**	**CSLI**
Actinic keratosis (ACK)	730	–	730
Basal cell carcinoma of skin (BCC)	845	291	1,136
Malignant melanoma (MEL)	52	118	170
Melanocytic nevus of skin (NEV)	244	147	391
Squamous cell carcinoma (SCC)	192	106	298
Seborrheic keratosis (SEK)	235	401	636
Total	2,298	1,063	3,361

### 3.2. Network structure

#### 3.2.1. Network structure

**Figure 2:**
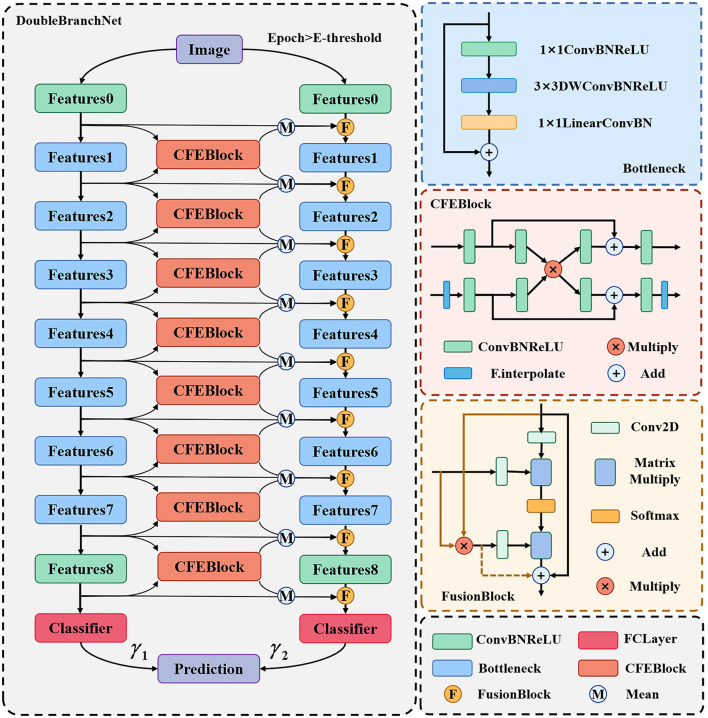
DBN structure diagram.

For the above CSLI dataset, we propose a two-branch classification network framework which can support the use of all feature extraction networks. Taking MobileNetV2 as an example, Figure 2 shows the overall structure of the network, which consists of two branches, the left side is called the “original network branch” and the right side is called the “fusion network branch.” The original network branch is responsible for the initial feature extraction of the input image, which is used to generate shallow features rich in texture and background information, as well as generative features rich in semantic information. The fusion network branch on the right uses FusionBlock to fuse the common features from the left side with the features output from the upper layer and pass them to the next layer, Bottleneck. The final results from the two branches are weighted and summed to calculate the final classification result. In addition, in order to ensure that the inability to generate more accurate feature maps on the left side at the early stage of training does not affect the training of the right side branch, we set a threshold E-threshold on epoch to ensure the quality of feature transfer, and when epoch < E-threshold, only the original network branch is trained, which is consistent with the original MobileNet. When epoch < E-threshold, only the original network branch is trained, at which time the network is consistent with the original MobileNet, and then the whole network is trained after the feature extraction ability of the original network on the dataset images reaches a certain level, which can ensure that the right fusion network branch can receive high-quality feature maps to improve the classification effect.

The results of the two branches in the above diagram are assigned different weights by the two learnable parameters γ_1_ and γ_2_. In addition, for the problem of inconsistent sizes of deep and shallow feature maps, we pre-scale the smaller feature maps in the CFEBlock and pass them on later. The structure of the CFEBlock and FusionBlock is described in detail in the following sections.

#### 3.2.2. Part of common features extraction

**Figure 3:**
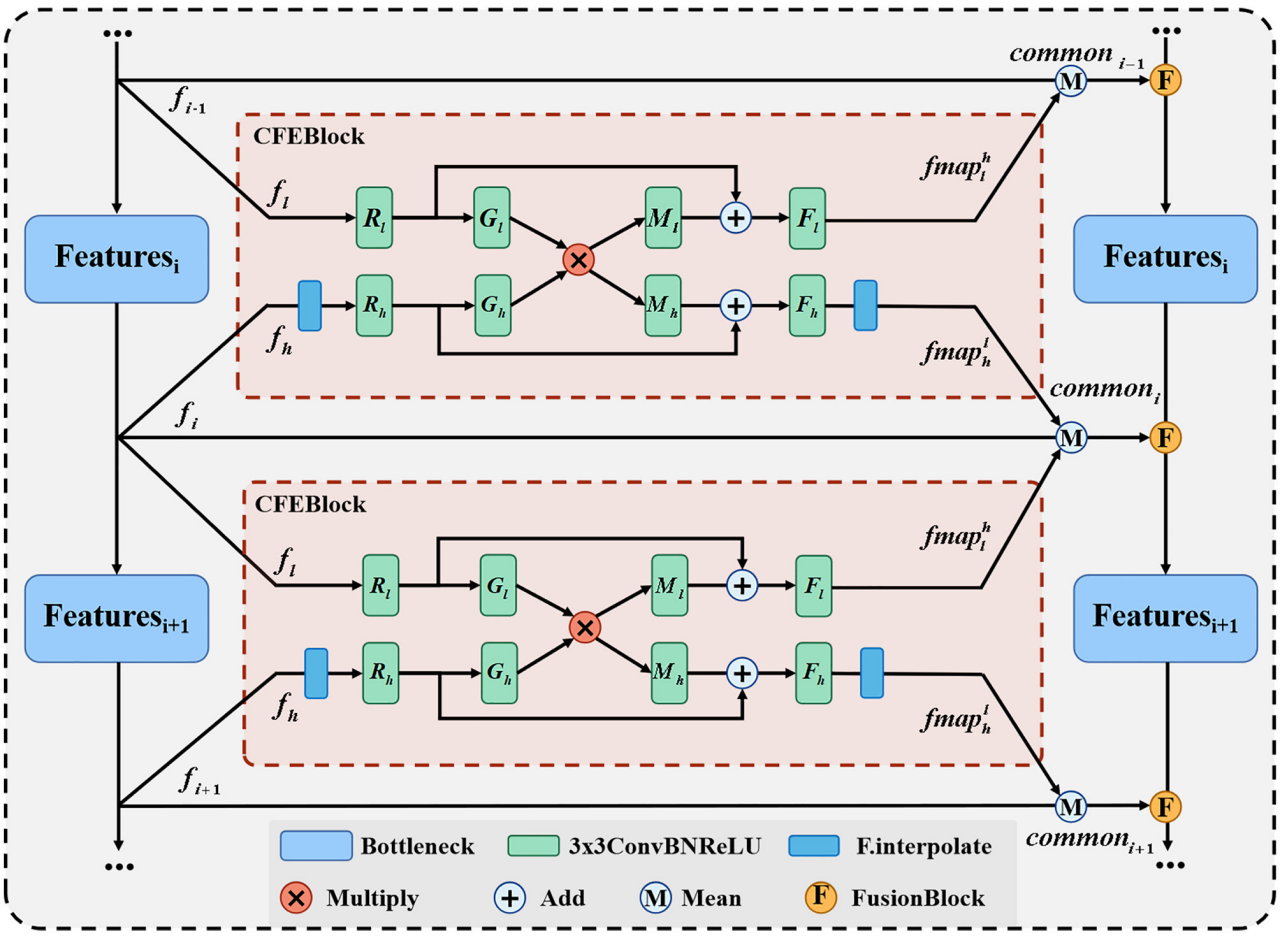
Network structure of CFEBlock.

Our proposed common feature extraction part uses CFEBlock as the base part to perform common feature extraction for the feature maps of adjacent layers, and then the feature maps of each layer of the original network branch are averaged with the common feature maps obtained after CFEBlock processing to obtain the final fused feature maps and passed to the corresponding layers of the fused network branch, and the detailed structure is shown in Figure 3.

We propose CFEBlock, inspired by the CFM module in (26). Compared with the CFM module, CFEBlock solves the problem of non-uniform input feature map channels and non-uniform output feature map sizes. For two feature maps with different input channels and sizes, CFEBlock uses convolution and upsampling operations to set the number of channels and size of the input feature map to the largest channel or size of the two feature maps, respectively. In the output, the number of channels and size of the two feature maps are restored using convolution and downsampling, and Equation (1) shows the specific operation flow of CFEBlock.


(1) $$CFEB(f_{l},f_{h})=\{\begin{array}{c} fmap_{l}^{h}=F_{l}(R_{l}(f_{l}))+M_{l}(G_{l}(R_{l}(f_{l}))\cdot G_{h}(R_{h}(f_{h})))\\ fmap_{h}^{l}=F_{h}(R_{h}(f_{h}))+M_{h}(G_{l}(R_{l}(f_{l}))\cdot G_{h}(R_{h}(f_{h}))) \end{array}$$

For ease of expression, we define the output of the CFEBlock as $fmap_{A}^{B}$, which takes the features of layer *A* of the original network branch as a reference and returns the branch of the same sizes and channels in layer *A* after common feature extraction with the feature map of layer *B*. Where *R*(·), *M*(·), *G*(·), *F*(·) are ConvBNReLU with convolution kernel of 3 × 3 and subscripts *l* and *h* denote the corresponding processing inputs *f*_*l*_ and *f*_*h*_. We use *CFEB*(·, ·) to denote the operation of CFEBlock, assuming that the input is $f_{l}\in R^{C_{1}\times W_{1}\times H_{1}}$ and $f_{h}\in R^{C_{2}\times W_{2}\times H_{2}}$, if the size of *f*_*h*_ is different from that of *f*_*l*_, then *f*_*h*_ is first upsampled to make it the same as *f*_*l*_ has the same width and height, the operation of *R*(·) will then *f*_*l*_ and *f*_*h*_ will channel number unified *C* = *Max*(*C*_1_, *C*_2_), and finally *F*_*l*_(·) and *F*_*h*_(·) will restore the channel number of the two layers to the size *C*1 and *C*2 at the input, respectively, and at the final output, if the initial size is different, the $fmap_{h}^{l}$ will be downsampled to make the two branches the same as at the input, i.e., $fmap_{l}^{h}\in R^{C_{1}\times W_{1}\times H_{1}}$ and $fmap_{h}^{l}\in R^{C_{2}\times W_{2}\times H_{2}}$.

We use CFEBlock as the base module of the feature fusion part, and input the feature map feature generated by each layer of the original network branch and the adjacent feature maps of the upper and lower layers to CFEBlock respectively to extract common features and obtain two common feature map *fmap*, and finally add the feature of the original network branch and *fmap* to find the average value to obtain the final common features, which is represented as shown in Equation (2).


(2) $$\begin{array}{l} common_{i}=Mean\left(f_{i},fmap_{i}^{i-1},fmap_{i}^{i+1}\right) \end{array}$$

In Equation (2), *common*_*i*_ denotes the result of the fusion of the i-th layer feature map, and *Mean*(·) denotes the average function.

#### 3.2.3. Part of feature fusion

**Figure 4:**
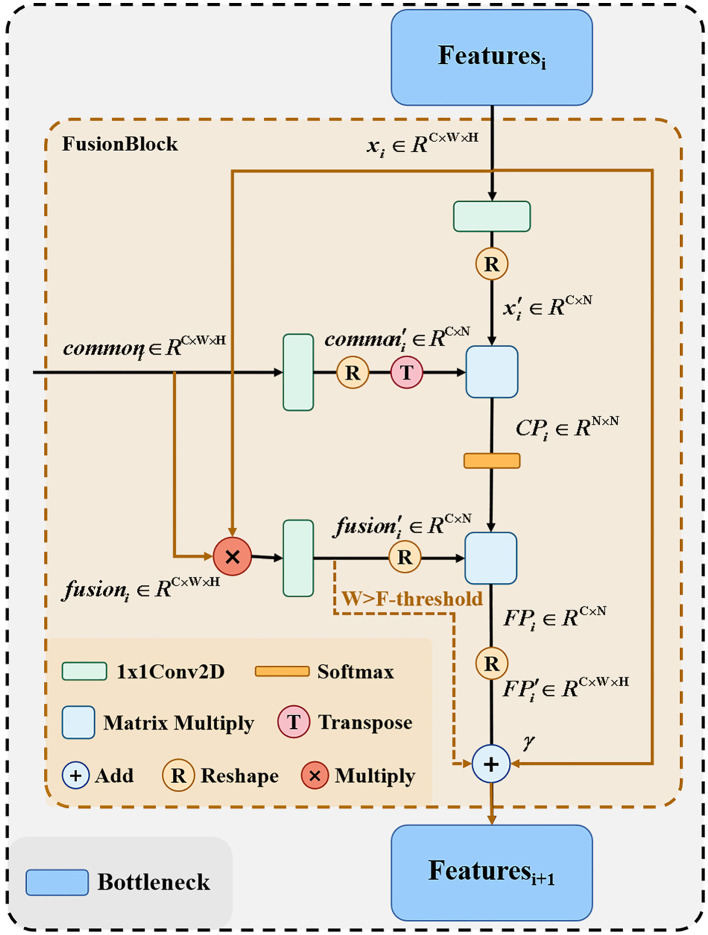
Network structure of FusionBlock.

In the common feature extraction section, the extracted common features from the original network branches and the adjacent layers need to be combined with the feature maps of the corresponding layers of the fusion branch network and passed to the next bottleneck layer. Figure 4 shows the detailed structure of the feature fusion part, because the matrix multiplication in the proposed method has certain requirements for computer arithmetic power, so for computers with different arithmetic power, we provide here two fusion processes, respectively, the solid line part and the colored line part of the figure, this part we control by the F-threshold, when the input feature size is greater than the F-threshold the module only go colored line part, when the feature size is less than or equal to the F-threshold, the module only go the solid line part.

After the feature fusion part from the original network branch extracts the common features between adjacent layers, the feature maps of the corresponding layers of the remaining fusion branch networks need to be combined and passed to the next layer bottleneck. For the output *x*_*i*_ of each layer of the fusion network branch and the common feature extraction part of each layer passing *common*_*i*_, we first perform the dot product operation on *x*_*i*_ and *common*_*i*_ to get *fusion*_*i*_, as shown in Equation (3).


(3) $$\begin{array}{l} fusion_{i}=x_{i}\cdot common_{i} \end{array}$$

Where *x*_*i*_, $common_{i},fusion_{i}\in R^{C\times W\times H}$. We then perform the reshape operation to merge the width and height of *x*_*i*_, *fusion*_*i*_, *common*_*i*_, respectively, and use 1 × 1 convolutional encoding to obtain $x_{i}^{\prime }$, $fusion_{i}^{\prime }$, $common_{i}\in R^{C\times N}\left(N=W\times H\right)$, after which the results of $x_{i}^{\prime }$ and $common_{i}^{\prime }$ encoding are subjected to matrix product operation to obtain the correlation map *CP*_*i*_, as shown in Equation (4).


(4) $$\begin{array}{l} CP_{i}={\left(common_{i}^{\prime }\right)}^{T}\times x_{i}^{\prime } \end{array}$$

Where $CP_{i}\in R^{C\times C}$, we obtain the fusion map *FP* after softmaxing the *CP*_*i*_ and matrix multiplying with $fusion_{i}^{\prime }$ as shown in Equation (5).


(5) $$\begin{array}{l} FP_{i}={\left(fusion_{i}^{\prime }\right)}^{T}\times Softmax(CP_{i}) \end{array}$$

By reshaping the obtained $FP_{i}\in R^{C\times N}$, we obtain the $FP_{i}^{\prime }\in R^{C\times W\times H}$. Finally, we train a learnable parameter γ as coefficients of $FP_{i}^{\prime }$ to be summed with the input *x*_*i*_ as the output $f_{i}^{\prime }$ of this part, as shown in Equation (6).


(6) $$\begin{array}{l} f_{i}^{\prime }=x_{i}+\gamma \cdot FP_{i}^{\prime } \end{array}$$

FusionBlock can calculate the correlation between *common*_*i*_ and *x*_*i*_ to obtain the hybrid graph $FP_{i}^{\prime }$. *x*_*i*_ can be summed with $FP_{i}^{\prime }$ to absorb common points from deep and shallow layers of the original network branches and highlight them in *x*_*i*_ as places to focus on when learning from the network. Since matrix multiplication after reshape requires arithmetic power greatly, we set the input features $f_{i}^{\prime }$ to be calculated as shown in Equation (7) when the image size is larger than F-threshold.


(7) $$\begin{array}{l} f_{i}^{\prime }=x_{i}+\gamma \cdot fusion_{i} \end{array}$$

In the above way, it is possible to fuse *common*_*i*_ and *x*_*i*_ while minimizing the computational power consumption.

## 4. Experimental setup and analysis of results

### 4.1. Dataset

We divided the CSLI dataset into a training set, a validation set, and a test set according to 8:1:1. During training, the model is trained on the training set and its performance is observed on the validation set. The model is finally tested in the test set. The split result is shown in Table 2.

**Table 2:** Number of diseases by category in the data set.

**Disease category**	**Train**	**Val**	**Test**	**Total**
Actinic keratosis (ACK)	584	73	73	730
Basal cell carcinoma of skin (BCC)	908	114	114	1,136
Malignant melanoma (MEL)	136	17	17	170
Melanocytic nevus of skin (NEV)	313	39	39	391
Squamous cell carcinoma (SCC)	238	30	30	298
Seborrheic keratosis (SEK)	508	64	64	636
Total	2,687	337	337	3,361

### 4.2. Evaluation indicators

The main metrics for the evaluation of automatic skin lesion image classification models are accuracy, precision, recall, specificity, f1score, receiver operating characteristic curve (ROC), area under curve (AUC), which are calculated from true positive (TP), true negative (TN), false positive (FP), false negative (FN), false positive rate (FPR), Balanced Accuracy (BA), and true positive rate (TPR):


(8) $$\begin{array}{l} Accuracy=\frac{TP+TN}{TP+TN+FP+FN} \end{array}$$

(9) $$\begin{array}{l} Precision=\frac{TP}{TP+FP} \end{array}$$

(10) $$\begin{array}{l} Recall=\frac{TP}{TP+FN} \end{array}$$

(11) $$\begin{array}{l} Specificity=\frac{TN}{TN+FP} \end{array}$$

(12) $$\begin{array}{l} F1Score=\frac{2\times Precision\times Recall}{Precision+Recall} \end{array}$$

(13) $$\begin{array}{l} FPR=\frac{FP}{TN+FP} \end{array}$$

(14) $$\begin{array}{l} TPR=\frac{TP}{TP+FN} \end{array}$$

(15) $$\begin{array}{l} BA=\frac{TPR+TNR}{2}=\frac{Recall+Specificity}{2} \end{array}$$

(16) $$\begin{array}{l} AUC=\overset{1}{\underset{0}{\int }}TPR\left(FPR\right)d\left(FPR\right) \end{array}$$

### 4.3. Analysis of experimental results

To address the problem of overfitting, in addition to modifying the network, we used L2 regularization to prevent excessive local weights when training the network using the loss function supervision and performed random vertical and horizontal flips on the image data. The output of the model is only a six-category confidence score. For the convenience of experimental description, we defined the DBN as “Small” when the F-threshold was chosen to be 0 and “Large” when the F-threshold was chosen to be 50, and added the E-threshold to the name, e.g., E-threshold = 25 and the DBN with F-threshold = 0 we call “DBN Small25.” The training set is only used for the training of the model, all quantitative analysis data are the results in the test set. Our experiments were all performed in a Python 3.7 environment, with the model using Pytorch as the architecture and single card training on 1 Tesla V100 GPU. The hyperparameters for the training are shown in Table 3.

**Table 3:** Training strategies.

**Parameters**	**Parameter setting**
Training set images	2,298
Validation set images	337
Test set images	337
Learning rate	0.0001
Batch	16
Epoch	100
Input size	[Batch size, 3, 384, 384 ]
Output size	[Batch size, 6]
Loss function	CrossEntropyLoss+L2 regularization
Optimizer	Adam
E-threshold	25, 50
F-threshold	0, 50

To verify the effectiveness of the DBN module, the following benchmark models are set to train the network according to the same training strategy, respectively: (1) the original MobileNetV2; (2) the common feature extraction part is removed, the feature fusion part is retained, and the feature maps of the corresponding layers of the original branch network and the fused branch network are directly fused using FusionBlock; (3) DBN Small25 (E-threshold = 25, F-threshold = 0); (4) DBN Small50 (E-threshold = 50, F-threshold = 0); (5) DBN Large25 (E-threshold = 25, F-threshold = 50); (6) DBN Large50 (E-threshold = 50, F-threshold = 50), and the results on the test set of CSLI are shown in Table 4, where the highest indicators are marked in bold.

**Table 4:** Ablation experiments.

**Model**	**Accuracy (%)**	**Precision (%)**	**Sensitivity (%)**	**Specificity (%)**	**BA (%)**	**F1 score (%)**	**AUC**
MobileNetV2 (14)	74.41	72.06	71.83	94.60	83.22	71.66	0.936
DBN fusion	76.24	77.67	71.18	94.27	82.72	74.28	0.931
DBN Small25	**79.52**	**77.83**	75.58	**95.63**	85.61	76.18	0.934
DBN Small50	77.94	77.11	**76.04**	95.27	**85.66**	**76.23**	**0.937**
DBN Large25	78.24	76.17	71.57	95.27	83.42	72.55	0.928
DBN Large50	77.06	73.15	73.88	95.16	84.52	73.37	0.925

The bold values identify the best indicators.

Compared with MobileNetV2, the accuracy of model prediction has been significantly improved after using the double-branch network; for DBN Fusion with CFEBlock removed, it can be seen from the table that precision has been significantly improved, but sensitivity has decreased to a certain extent, indicating that FusionBlock is helpful to improve precision. Although it is impossible to observe the effect of CFEBlock alone by removing FusionBlock alone (because the features cannot be added or multiplied directly to achieve a good fusion effect, resulting in a decrease in the composite index), DBN Large25 can reflect the improvement of sensitivity after adding CFEBlock on top of DBN Fusion and the improvement of DBN Small25 on features. However, DBN Large25 can reflect the improvement of sensitivity after adding CFEBlock on top of DBN Fusion and DBN Small25 can reflect CFEBlock can help to improve sensitivity to a certain extent. Comparing MobileNetV2 with DBN Fusion and DBN Small25 with DBN Large25, it shows that the process of FusionBlock solid line affects sensitivity to some extent. Comparing the changes in accuracy, precision, and sensitivity in DBN Small25 and DBN Small50 and DBN Large25 and DBN Large50 suggests that the E-threshold should not be too large in its selection, and that too large a gap between the two branches may also be detrimental to the learning of the network. From the overall table, DBN Small25 has a better overall index for our proposed CSLI dataset, but this may also be caused by the small sample of the dataset.

The comparison of our proposed DBN Small25 with other classical classification models on the CSLI test set is shown in Table 5, and it can be seen that our proposed DBN network has better performance on the clinical dataset CSLI.

**Table 5:** Comparison of DBN and different classification network models on the CSLI test set.

**Model**	**Accuracy (%)**	**Precision (%)**	**Sensitivity (%)**	**Specificity (%)**	**BA (%)**	**F1score (%)**	**AUC**
DenseNet121 (7)	76.76	73.78	73.35	95.00	84.17	73.37	0.935
ResNet50 (11)	77.35	74.97	72.45	95.12	83.79	73.26	0.933
MobileNetV2 (14)	74.41	72.06	71.83	94.60	83.22	71.66	**0.936**
EfficientNetb0 (27)	76.18	73.59	72.03	94.84	83.44	72.46	0.929
EfficientNetb4 (27)	76.76	75.20	71.47	94.98	83.22	72.53	0.927
EfficientNetb7 (27)	79.18	**77.88**	75.18	95.51	85.35	75.68	0.934
DBN Small25	**79.52**	77.83	**75.58**	**95.63**	**86.22**	**76.18**	0.934

The bold values identify the best indicators.

We tested these models again on the publicly available dataset HAM10000, and the results in Table 6 shows that our proposed model outperforms in terms of sensitivity, balanced accuracy, f1score, and AUC.

**Table 6:** Comparison of DBN and different classification network models on HAM10000.

**Model**	**Accuracy (%)**	**Precision (%)**	**Sensitivity (%)**	**Specificity (%)**	**BA (%)**	**F1score (%)**	**AUC**
DenseNet121 (7)	**91.86**	86.64	83.62	**97.61**	90.62	84.90	**0.985**
ResNet50 (11)	90.51	86.35	81.82	97.32	89.57	83.49	0.980
MobileNetV2 (14)	91.06	87.09	80.21	97.44	89.57	83.20	0.983
EfficientNetb0 (27)	91.31	**87.31**	82.24	97.58	89.91	84.43	0.984
EfficientNetb4 (27)	90.61	83.88	82.30	97.28	89.79	82.44	0.982
EfficientNetb7 (27)	91.65	86.10	83.85	97.50	90.68	84.81	0.983
DBN Small25	91.58	85.89	**84.20**	97.58	**90.89**	**85.03**	**0.985**

The bold values identify the best indicators.

Table 7 shows the comparison between the proposed DBN model and other models on the HAM10000, and the results show that the DBN is better at classification.

**Table 7:** Comparison of DBN with other algorithms.

**References**	**Method**	**Accuracy (%)**
Khan et al. (28)	Mask R-CNN and 24-layered CNN	86.5
Pacheco and Krohling (24)	CNN fusion segmentation network and 30-layered CNN	87.02
Srinivasu et al. (15)	MobileNetV2, LSTM	90.21
Afza et al. (29)	Three-step superpixel segmentation and ResNet50, Naïve Bayes classifier	85.50
Wang et al. (30)	G-DMN	87.07
Ours	DBN	91.58

Table 8 shows the classification of all disease categories in the CSLI test set by DBN Small25. Of all six disease categories, DBN was a poor indicator for MEL and SCC, which may be due to the small sample size of the MEL and SCC datasets resulting in the model not being able to fully learn the features of MEL and SCC, and the fact that MEL and SCC have a high visual similarity to other diseases, and therefore when classifying the two disease categories of MEL and SCC, the network tended to predict the results as being more similar to them more similar and to the disease with a larger sample size. However, by analyzing the AUC values, it was found that the AUC values for MEL were not low, indicating that the cut-off values selected by the model in the table for MEL prediction were not the best cut-off values, but in fact the AUC combined the predictive performance of all the cut-off values, and for the low MEL metric and high AUC indicated that this was due to a high bias in the sample (i.e., the similar NEV category with a high sample size influenced the model's judgement of MEL), rather than a poorer ability of the model to predict MEL. For SCC diseases, the lower metrics and AUCs suggest that the model has a greater problem in predicting this category of disease. By looking at the dataset, it was found that SCC had a high similarity to the three disease categories ACK and BCC, but the training sample sizes for ACK and BCC were more than twice or three times larger than SCC, respectively. The difference in data size and the high similarity of the disease images led to the model's ability to show relatively poor results. In contrast, for other types of diseases, the model predicted better than the composite index.

**Table 8:** DBN Small25 classification indicators for each type of disease in the CSLI test set.

**Disease category**	**Accuracy (%)**	**Precision (%)**	**Sensitivity (%)**	**Specificity (%)**	**BA (%)**	**F1score (%)**	**AUC**	**No. of samples**
Actinic keratosis (ACK)	–	71.60	79.45	91.29	85.37	75.32	0.921	73
Basal cell carcinoma of skin (BCC)	–	84.11	78.95	92.38	85.67	81.45	0.915	114
Malignant melanoma (MEL)	–	76.92	58.82	99.06	82.41	66.67	0.966	17
Melanocytic nevus of skin (NEV)	–	78.26	92.31	96.64	94.48	84.71	0.976	39
Squamous cell carcinoma (SCC)	–	69.56	53.33	97.72	75.53	60.38	0.846	30
Seborrheic keratosis (SEK)	–	86.56	90.63	96.70	93.67	88.55	0.981	64
Average	79.52	77.83	75.58	95.63	86.22	76.18	0.934	56

**Figure 5:**
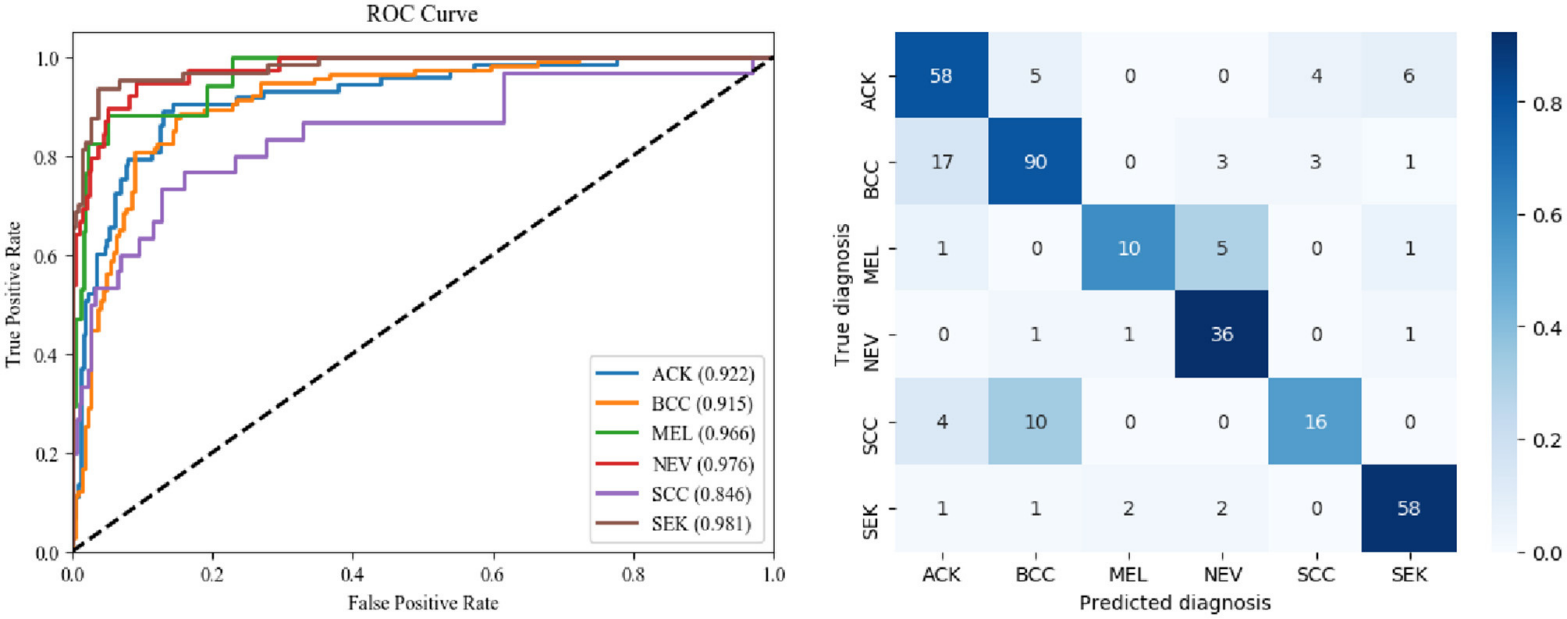
ROC curve and confusion matrix of DBN.

The ROC curves for DBN and the confusion matrix for the six categories in the CSLI test set are shown in Figure 5. the closer to the top left of the ROC curve indicates better model performance, and the information reflected in Figure 5 is similar to that in Table 8. the DBN performs poorly on SCC, but the AUC for MEL disease is not low, and the combination of the confusion matrix shows that the main reason for the low accuracy of MEL is that the data set The sample was too small and there were many disease categories. The prediction results of a single sample have a greater impact on the overall index of the disease category. From the confusion matrix, the error in SCC is essentially caused by the confusion of the prediction results with BCC. From the images of the dataset sample, SCC and BCC are similar, and the BCC category accounts for nearly 1/3 of the total dataset, which is why the model tends to predict SCC as BCC when it encounters similar looking diseases.

We extracted images collected from the CSLI as a dataset and compared the DBN model's predictions for the original hospital images and the cropped images after data processing for the metrics that were calculated on the test set, and the results are shown in Table 9.

**Table 9:** Training DBN results with uncropped and centrally cropped images.

**Model**	**Accuracy (%)**	**Precision (%)**	**Sensitivity (%)**	**Specificity (%)**	**BA (%)**	**F1score (%)**	**AUC**
Uncropped	81.01	85.10	**75.83**	94.47	85.16	78.14	0.930
Centercropped	**82.27**	**85.48**	75.69	**94.86**	**85.28**	**78.34**	**0.933**

The bold values identify the best indicators.

The results showed essentially the same results using uncropped images, which is because DBN incorporates information from deeper feature maps that contain richer semantic information, such as the location or size of the lesion, when recognizing images, so that the model still has some recognition ability even if the lesion is not in the center. However, a centrally cropped dataset is more useful for training the model because lesions in the PAD-UFES-20 dataset are basically in the center, and a centrally cropped dataset ensures a more uniform distribution of the CSLI data samples. Secondly, some disease lesions are too small and scaled up when fed into the network, which may lose too much information and cause the classifier to learn the wrong information. Therefore, centering before scaling still helps the training of the classifier, and as shown in the table, the metrics of the model trained using the centered cropped dataset are still slightly higher than those of the uncropped dataset.

## 5. Conclusion

In this paper, we propose a new dataset CSLI and a double-branch network structure DBN for this dataset, which contains two identical branches of the feature extraction network, and this structure makes the feature map of the shallow layer of image classification can be bi-directional with the deep layer feature map for information transfer between them, with higher flexibility and accuracy. It is not limited to using two identical structures of MobileNetV2 as two branches in this paper, but it is also feasible to use other different feature extraction networks as one of the branches, or to use multiple branches at the same time like random forests, and to perform common feature extraction and fusion of feature maps on this basis, which is like several skin disease specialists analyzing the disease and coming to their own conclusions, and then he treating doctor takes into account the results of all and combines them with the views they give to make a combined judgement to reach a conclusion with higher accuracy and stability.

Our proposed DBN consists of two important components, namely the common feature extraction part and the feature fusion part, which are demonstrated to be effective on the CSLI dataset in the experiments in Section 4.3. The CFEBlock module of the common feature extraction part can combine the shallow and deep information and mention the common features between the two feature maps, while the metrics reflect the Sensitivity improvement. In the feature fusion part, we give two different fusion routes, in the experiments, the fusion scheme which consumes less arithmetic power has a greater improvement on Sensitivity, but may affect Precision, while the other strategy contains matrix multiplication fusion scheme by calculating the correlation coefficient between feature maps, which can have a more accurate localization of diseases, and basically does not affect By comparing with other models and analyzing the ROC curve and confusion matrix, DBN is better than other classical skin classification models in classifying the comprehensive performance of skin diseases, although there are some difficulties in classifying SCC and MEL.

Although DBN has achieved some results in the experiments, it still has some limitations. Firstly, the unbalanced nature of the dataset categories causes DBN to present a higher AUC and lower accuracy in predicting several categories of MEL. When predicting BCC and SCC images with high similarity, DBN will preferentially predict the results to the BCC category with higher sample size. For the problem of DBN's low prediction on cancerous lesions, the reason is caused by the uneven sample distribution of the dataset. This is because the sample size of the images of cancer patients itself is too small and therefore the model cannot give a high confidence level when judging these images, whether it is a DBN model or any other model on this dataset. This problem can be solved by setting a Top-Accuracy and confidence threshold, i.e., the model prioritizes the results as cancer when the chance of the disease category being predicted as cancer is greater than a set threshold. The final prediction can also be obtained by setting different importance factors for each category of disease, and multiplying the probability of each category of disease by the importance factor after the results are obtained. Both of these approaches can solve the problem of low sensitivity of cancer-like diseases. We will also focus on improving the collection of samples related to cancer diseases in our subsequent studies and data set collection to reduce the impact of data imbalance on the classification model.

In addition, DBN uses multiple feature extraction networks and matrix multiplication operations in FusionBlock, which has high requirements on computer arithmetic power, so in the subsequent study, we will try to reduce the DBN parameter size and arithmetic power requirements. For example, through experiments, we will filter and retain some of the CFEBlocks that have a greater impact on the classification results, more lightweight branches and feature fusion strategies that consume less arithmetic power, so as to ensure that the DBN can significantly reduce the model size while retaining the accuracy of clinical skin images, and thus can be embedded into mobile devices to facilitate disease prediction.

As a black box, deep neural networks lack interpretability and robustness, making them vulnerable to adversarial attacks. When training data is disturbed, their behavior and performance may encounter problems (31). The classification results of a dual branch network are determined jointly by the two branches and have a certain degree of robustness. In subsequent research work, experiments such as noise immunity will be added to optimize the stability and robustness of the model, such as adding anti-interference branches to the model, or randomly adding noise or dropout some weights of layers to the training dataset, in order to improve the stability of the network.

In addition to the problems with the model described above, the following problems remain in the dataset: in studies of clinical images, the images taken do not have a stable color interval, i.e., color constancy, like dermoscopic images, due to differences in the environment, and clinical skin images taken in different locations and at different times are highly variable, as Salvi et al. (32) and Ng et al. (33). As for the CSLI dataset, we will continue to collect data from hospitals to further expand this dataset, and in subsequent studies we will try not to artificially crop the focal areas, but to modify the network structure so that the network can give higher attention to the focal areas.

## Data availability statement

The datasets for this study are not available due to patient confidentiality and data privacy. PAD-UFES-20 data are available at this link: https://data.mendeley.com/datasets/zr7vgbcyr2/1. The data that support the findings of this study are available from the corresponding author upon reasonable request.

## Ethics statement

The studies involving human participants were reviewed and approved by the Second Hospital of Jilin University. The patients/participants provided their written informed consent to participate in this study.

## Author contributions

QQ was the main author of the thesis, proposing the model ideas and designing the implementation of the method to write the thesis. HW was the lead author's MSc supervisor who provided insightful analytical ideas, suggestions, comments, and feedback that greatly improved the thesis and the experimental ideas. XL and CY, who are responsible for being doctors in the hospital's dermatology department, provided us with clinical images and GroundTruth, and helped us construct the CSLI dataset. WS provided comments and feedback on the paper and asked some questions about the proposed methodology and how it could be improved. All authors contributed to the article and approved the submitted version.
